# The relationship between in-hospital location and outcomes of care in patients diagnosed with dementia and/or delirium diagnoses: analysis of patient journey

**DOI:** 10.1186/s12877-016-0372-5

**Published:** 2016-11-24

**Authors:** Lua Perimal-Lewis, Clare Bradley, Paul H. Hakendorf, Craig Whitehead, Louise Heuzenroeder, Maria Crotty

**Affiliations:** 1 Rehabilitation, Aged and Extended Care, Flinders University, GPO Box 2100, 5001 Adelaide, South Australia Australia; 2 Flinders Medical Centre & Flinders University, Adelaide, South Australia Australia; 3 SA Health, Adelaide, South Australia Australia; 4 NHMRC Partnership Centre on Dealing with Cognitive and Related Functional Decline in Older People, Adelaide, Australia

**Keywords:** Dementia and/or delirium, Health care delivery, Public hospitals, Patient journey, Outcome and process assessment (healthcare), Ward outliers

## Abstract

**Background:**

The discrepancy between the number of admissions and the allocation of hospital beds means that many patients admitted to hospital can be placed in units or wards other than that which specialise in the patient’s primary health issue (home-ward). These patients are called ‘outlier’ patients. Risk factors and health system outcomes of hospital care for ‘outlier’ patients diagnosed with dementia and/or delirium are unknown. Therefore, the aim of this research was to examine patient journeys of people with dementia and/or delirium diagnoses, to identify risk factors for ‘inlier’ or ‘outlier’ status and patient or health system outcomes (consequences) of this status.

**Methods:**

A retrospective, descriptive study compared patients who had dementia and/or delirium according to the proportion of time spent on the home ward i.e. ‘inliers’ or ‘outliers’. Data from the patient journey database at Flinders Medical Centre (FMC), a public hospital in South Australia from 2007 and 2014 were extracted and analysed. The analysis was carried out on the patient journeys of people with a dementia and/or delirium diagnosis.

**Results:**

When 6367 inpatient journeys with dementia and/or delirium within FMC were examined, the Emergency Department (ED) Length of Stay (LOS) after being admitted as inpatient was prolonged for ‘outlier’ patients compared to ‘inlier’ patients (OR: 1.068, 95% CI: 1.057–1.079, *p* = 0.000). However, the inpatient LOS for’outlier’ patients was only marginally shorter than that of the ‘inlier’ patients (OR: 0.998, 95% CI: 0.998–0.998, *p* = 0.000). The chances of dying within 48 h of admission increased for ‘outlier’ patients (OR: 1.973, 95% CI: 1.158–3.359, *p* = 0.012) and their Charlson co-morbidity Index was higher (OR: 1.059, 95% CI: 1.021–1.10, *p* = 0.002). Completion of discharge summaries within 2 days post-discharge for ‘outlier’ patients was compromised (OR: 1.754, 95% CI: 1.492–2.061, *p* = 0.000).Additionally, ‘outlier’ patients were more likely to be discharged to another hospital for other care types not offered at FMC (OR: 1.931, 95% CI: 1.559–2.391, *p* = 0.000).

**Conclusion:**

An examination of the patient journeys at FMC has determined that the health system outcomes for patients with dementia and/or delirium who are admitted outside of their home-ward are affected by in-hospital location despite the homogenous nature of the study population.

## Background

Dementia and/or delirium affect a growing number of older adults. In Australia, dementia is projected to remain the third leading cause of disability burden and fourth leading cause of overall burden of disease until at least 2020 [1]. In 2011, about 9% of Australians aged 65 years and over had dementia [2]. Patients with dementia have an increased risk of delirium and dementia is often a comorbid condition for delirium [3–6]. Delirium is also common in hospitalised elderly patients [3]. Distinction between dementia and delirium in older people is complicated as dementia co-occurs in most of delirium cases [5, 7]. On the other hand, it is also probable for patients with delirium to be misdiagnosed as having dementia [8]. The projected increase in the older population, many of whom have been diagnosed with dementia, increases the demand for care in public hospitals [9].

Australian public hospitals often work at high levels of bed occupancy, generally between 95 and 100% [10]. As a consequence, it is often difficult to admit patients into the home-ward that corresponds to the clinical team managing the patient’s primary health care need. Thus patients may have to spend periods of time in outlier wards until a suitable bed becomes available in their home-ward [10]. This disrupts both ward-based and team-based models of care which may lead to poor quality of care [10].

A recent study found that there was a strong association between time spent in outlier wards and the number of emergency calls to Medical Emergency Teams (METs). The study used MET activation as a measure for adverse events [11]. Other studies have shown that, patients admitted to outlier wards (*surgical patients on medical ward*) are prone to medication errors [12], prolonged hospital Length of Stay (LOS) for patients with heart failure [11], greater in-hospital mortality and lower rates of discharge summary completion [10]. Being an inpatient in a hospital can be a confusing experience and harmful for a person with dementia [13]. Dementia patients in acute hospitals are also known to receive inadequate or inappropriate quality care [14]; dementia patients with palliative care needs are less frequently referred to palliative care teams and receive fewer palliative medications [15]. In addition, the Australian Government’s prescribed target for a patient’s LOS in the Emergency Department (ED) is less than 4 h likely increases the frequency of patients being sent to outlier wards [1, 10]. Patients in outlier wards maybe be transferred to the appropriate home-ward when such ward becomes available. Numerous ward movements may be detrimental to the health of elderly patients. Moving elderly patients at hospital has been associated with an increased incidence of delirium [16].

Elderly dementia and/or delirium patients with extended LOS in outlier wards and receiving fragmented care delivery are at risk of poor hospital outcomes. There are no known studies done so far on the effects of a patients’ outlier ward status on hospital outcome measures for dementia and/or delirium patients. Therefore the aim of this study is to identify the risk factors for ‘inlier’ or ‘outlier’ status and patient or health system outcomes of this status both during and after their hospital admission.

## Methods

### Study design and setting

All patients admitted to the Flinders Medical Centre (FMC), who had been coded with a dementia and/or delirium diagnoses in the hospital separation data for the period 2007–2014, were included in the study. FMC is a 500 bed public teaching hospital in South Australia with approximately 40,000 ED presentations per annum. FMC’s inpatient services comprise of short-stay and long-stay units with defined home-ward location. A home-ward is defined as the ward where the multidisciplinary team primarily responsible for the care of a particular patient is located [10]. The wards that were home-wards were operationally defined throughout the period of observation. The computerised bed management system used at FMC instantaneously updates its information pertaining to allocated home-wards for each specialist unit according to their continuously updated business rules. Accordingly the ward inlier or outlier status of a patient admitted to a ward is also automatically reflected and considerable care is taken by FMC staff to keep the relevant data updated to the specialist team responsible for delivery of care. Furthermore, the percentage of patients who were outliers was a regularly reported hospital indicator as such this report relies on accurate information on ward inlier or ward outlier status [10].

If a patient’s home-ward is not available at admission, the patient might be housed in outlier ward/s including ‘boarding’ in the ED for a period of time until an outlier ward or the home-ward becomes available. During the time when patients are housed in outlier ward/s, the care responsibilities remain with the home-ward team allocated to the care of the patient. As such, any amount of time spent away from the patient’s home-ward is considered as outlier time by the hospital.

FMC also has an Acute Medical Unit (AMU) comprising of 30 beds. The AMU admits all General Medicine (GM) patients and Acute Care of the Elderly (ACE) patients where; (i) short-stay patients are cared for by the AMU team, in which case, the AMU is a home-ward for these patients; (ii) patients are housed temporarily in the AMU whilst the AMU team allocates an appropriate long-stay team, therefore the home-ward designation for these patients will eventually change; and (iii) patients who require initial cardiac monitoring and closer observation for a short time are housed temporarily at the AMU prior to their admission to long-stay home-wards.

### Data

Hospital ED presentations from 1/01/2007 to 22/09/2014 for patients with a dementia and/or delirium diagnoses based on the International Classification of Diseases, Tenth Revision, Australian Modification (ICD-10-AM) recorded as either the principal diagnosis and/or included as part of the 24 additional diagnoses were extracted from FMC’s ED database. The ICD-10-AM codes used are listed in Table 1. The relevant inpatient data were extracted from FMC’s in-house ED database retrospectively and were linked to FMC’s patient journey database to extract information on ward movements for each patient from their time of admission until discharge. The FMC patient journey database is a continuously updated extract of the hospital’s patient admission and tracking databases. All inpatient movements between wards and between units are time-stamped and recorded. The FMC patient journey database also records post-discharge deaths extracted from the register of births, deaths and marriages.

**Table 1:** Dementia and/or delirium ICD-10-AM codes

Dementia codes
F00.0* Dementia in Alzheimer’s disease with early onset (G30.0^†^)
F00.1* Dementia in Alzheimer’s disease with late onset (G30.1^†^)
F00.2* Dementia in Alzheimer’s disease, atypical or mixed type (G30.8^†^)
F00.9* Dementia in Alzheimer’s disease, unspecified (G30.9^†^)
F01.0 Vascular dementia of acute onset
F01.1 Multi-infarct dementia
F01.2 Subcortical vascular dementia
F01.3 Mixed cortical and subcortical vascular dementia
F01.8 Other vascular dementia
F01.9 Vascular dementia, unspecified
F02.1* Dementia in Creutzfeldt-Jakob disease (A81.0^†^)
F02.2* Dementia in Huntington’s disease (G10^†^)
F02.3* Dementia in Parkinson’s disease (G20^†^)
F02.4* Dementia in human immunodeficiency virus [HIV] disease (B22^†^)
F02.8* Dementia in other specified diseases classified elsewhere
Dementia (in):
• cerebral lipidosis (E75.-^†^)
• epilepsy (G40.-^†^)
• hepatolenticular degeneration (E83.0^†^)
• hypercalcaemia (E83.5^†^)
• hypothyroidism, acquired (E01.-^†^, E03.-^†^)
• intoxications (T36-T65^†^)
• Lewy body disease (G31.3^†^)
• multiple sclerosis (G35^†^)
• neurosyphilis (A52.1^†^)
• niacin deficiency [pellagra] (E52^†^)
• polyarteritis nodosa (M30.0^†^)
• systemic lupus erythematosus (M32.-^†^)
• trypanosomiasis (B56.-^†^, B57.-^†^)
• uraemia (N18.5^†^)
• vitamin B12 deficiency (E53.8^†^)
F03 Unspecified dementia
Delirium Codes
F05.0 Delirium not superimposed on dementia, so described
F05.1 Delirium superimposed on dementia
F05.8 Other delirium
F05.9 Delirium, unspecified

*The asterisk symbol denotes a code describing the manifestation of a disease and should always be assigned together with the appropriate aetiology code

^†^The dagger symbol denotes a code describing the aetiology or underlying cause of a disease and should always be assigned together with the appropriate manifestation code

**Fig. 1:**
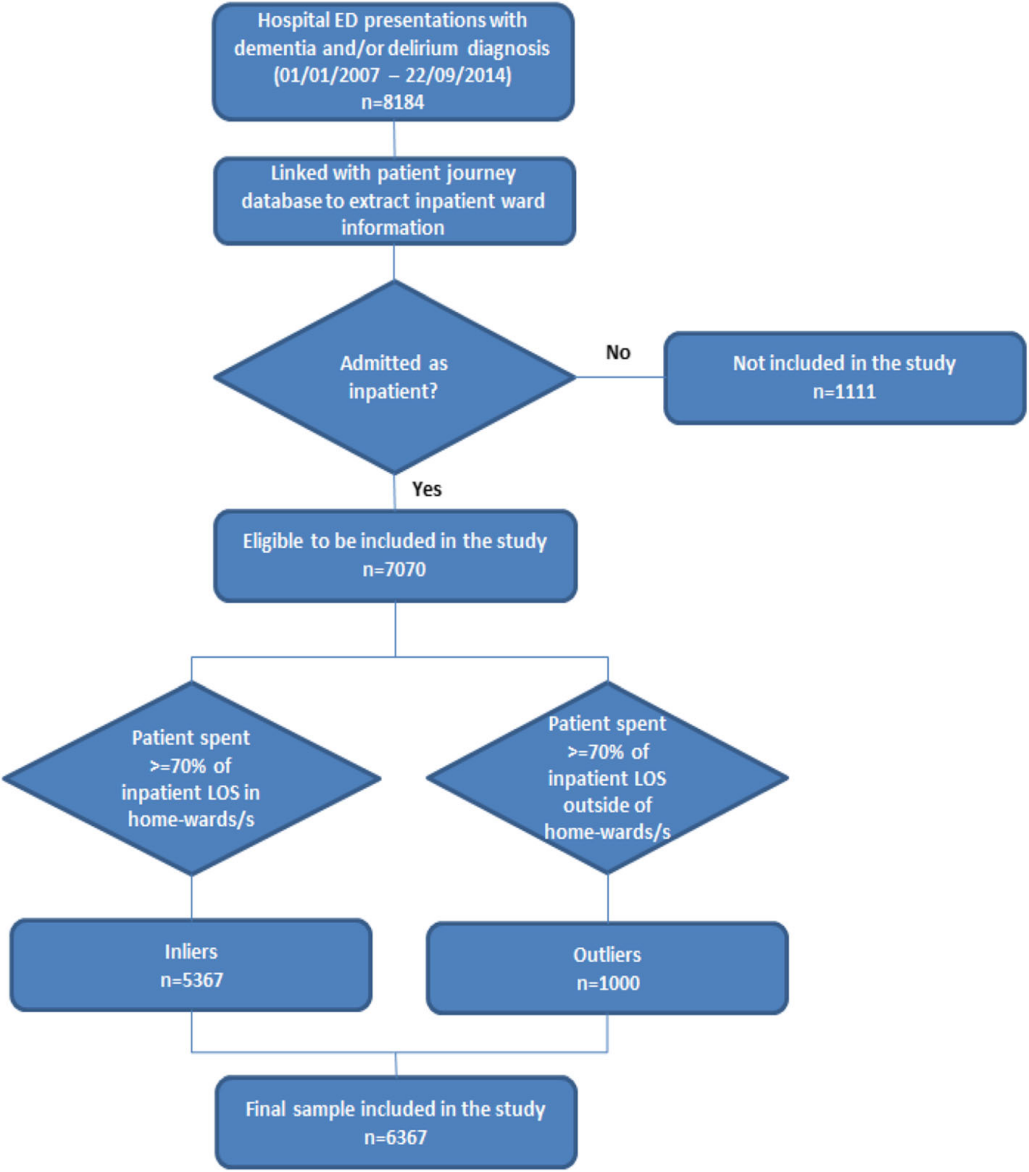
Flowchart of data inclusion and exclusion

The initial data extraction of 8184 records of individual patients from the hospital’s ED database was merged with data from the hospital’s patient journey database using deterministic record linkage methods. Deterministic linkage requires an exact match between the identifying variables for data to be attributed to the same individual (as opposed to probabilistic linkage which can more easily account for some variation in the identifying data) [17]. A total of 1111 in-scope ED presentations were not admitted as inpatients and therefore their records were not present in the patient journey database and so were excluded from the analysis, giving a new sample size of 7073 patient records. Three patient journeys were further excluded from the sample because of incomplete values and the absence of valid inpatient ward records, giving a possible sample size of 7070 patients. Inpatient LOS (hospital stay) is the difference between date/time of admission and date/time of discharge. An admitted patient may spend part or all of their hospital stay in a home-ward. Admitted patients generally move from an outlier ward to a home-ward. If the patient spent > =70% of their hospital stay in home-ward/s, we defined their status for the purposes of this study as an ‘inlier’ patient. If the patient spent > =70% of their hospital stay outside their home-ward, we defined their status as an ‘outlier’ patient. This > =70% threshold captured 90% of the study population leading to a final sample size of 6367. The process of data inclusion and exclusion is depicted in the flow diagram (Fig. 1). A > =70% threshold was also used to determine inlier versus outlier classification based on a previous study [10].

#### Dependent variable

The dependent variable was the status of a patient as ‘outlier’ patient versus ‘inlier’ patient as defined for the purpose of this study. Accordingly, those patients spending > =70% of their hospital stay in outlier ward/s were classified as outliers and those patients spending > =70% of their hospital stay in home-ward/s were classified as inliers. This dependent variable is a process related outcome. Process measures are used to assess delivery of care and coordination of care to improve patient health related measures [18].

#### Independent variables

Independent variables used to adjust for available confounders in the logistic regression model were: age; inpatient LOS; total time spent in the ED after admission; nature of separation (*home, other hospital–up transfer, other hospital–down transfer, nursing home or hostel, in-hospital mortality, other types of discharges*); in-hospital mortality within 48 h of admission; if discharge summaries were sent within 2 days of discharge; episode of care (*acute and non-acute*); if patient died within 28 days of discharge; sex; principal diagnosis of dementia (*yes, no*); Charlson co-morbidity index [19]; source of referral (*Other or unknown, other private medical practise, residential aged care facilities, inter-hospital transfer, outpatient department, casualty/emergency*); and treatment priorities (*Australasian Triage Scale (ATS) 1–5, booked/elective patients, not assigned*). The variables age, sex, principal diagnosis of dementia and Charlson co-morbidity index are risk factors, whereas the other variables are health system outcomes.

### Statistical analysis

Descriptive statistical analyses of patient demographics were performed. Categorical variables were compared using Chi-squared tests, and continuous variables were compared using Wilcoxon-Mann–Whitney tests. Treatment priority; episode of care (Table 2); discharged from outlier ward (number of patients discharged from an outlier ward) and admission decision made inside of working hours (Table 3) were compared using the two-sample test of proportions. Inside working hours are from 0800 to 1800. Logistic regression model was used to analyse the risk factors and health system outcomes where these potential confounders of clinical interest were included as independent variables in comparing the adjusted odds ratio of ‘outlier patients’ versus ‘inlier patients’ with respect to the variable. A *p* value of <0.05 was considered as statistically significant. The Charlson co-morbidity index [18] as modified by Quan and colleagues [20] was calculated for all patients. The logistic regression model was assessed using Hosmer and Lemeshow’s goodness-of-fit test. In addition a sensitivity analysis was undertaken to assess the impact of a different definition of the dependent variable [21]. The purpose of the sensitivity analysis was to assess the robustness of the results. The decision on the cut-off point used in the sensitivity analysis was done in consultation with senior doctors and hospital epidemiologists who understand the real-world nature of these data. For the sensitivity analysis; if the patient spent 100% of their hospital stay in home-ward, we defined their status as an ‘inlier’ patient. If the patient spent 100% of their hospital stay outside their home-ward, we defined their status as an ‘outlier’ patient. This reduced the eligible cohort to 1269 (18%). Statistical analyses were performed using STATA 13.0 (StataCorp., College Station, TX, USA).

**Table 2:** Patient characteristics

	Total (*n* = 6367)	Inlier (*n* = 5367, 84.29%)	Outlier (*n* = 1000 15.71%)	*p*-value
Demographics				
Age, mean (SD)	81.07 (11.48)	80.83 (11.72)	82.39 (9.98)	0.0006
Charlson Index, mean (SD)	2.03 (2.06)	2.03 (2.07)	2.05 (1.96)	0.1534
Female, n (%)	3563 (55.96)	3005 (55.99)	558 (55.80)	0.9114
Principal Diagnosis coded with Dementia, n (%)	725 (11.39)	597 (11.12)	128 (12.8)	0.1254
Treatment Priority				
ATS 1, n (%)	342 (5.37)	296 (5.52)	46 (4.60)	0.2386
ATS 2, n (%)	1282 (20.14)	1071 (19.96)	211 (21.10)	0.4072
ATS 3, n (%)	2982 (46.84)	2474 (46.10)	508 (50.80)	0.0062
ATS 4, n (%)	1194 (18.75)	998 (18.60)	196 (19.60)	0.4548
ATS 5, n (%)	61 (0.96)	44 (0.82)	17 (1.70)	0.0087
ATS 6, n (%)- *(Not triaged)*	52 (0.82)	51 (0.95)	1 (0.10)	0.0061
Not assigned, n (%)	454 (7.13)	433 (8.07)	21 (2.10)	0.0000
Episode of Care				
Acute, n (%)	6225 (97.77)	5246 (97.75)	979 (97.90)	0.7613
Non-Acute, n (%)	142 (2.23)	121 (2.25)	21 (2.10)	0.7613
Nature of Separation				
Home, n (%)	3664 (57.55)	3117 (58.08)	547 (54.70)	0.0473
Other hospital – up transfer, n (%)	621 (9.75)	445 (8.29)	176 (17.60)	0.0000
Nursing home or hostel, n (%)	516 (8.10)	449 (8.37)	67 (6.70)	0.0763
In-hospital mortality, n (%)	520 (8.17)	424 (7.90)	96 (9.60)	0.0715
Other hospital – down transfer, n (%)	973 (15.28)	873 (16.27)	100 (10.00)	0.0000
Other types of discharge, n (%)	73 (1.15)	59 (1.10)	14 (1.40)	0.4122

**Table 3:** Health system outcomes (or consequences) variables

	Total (*n* = 6367)	Inlier (*n* = 5367, 84.29%)	Outlier (*n* = 1000 15.71%)	*p*-value
Time spent boarding in ED (Hrs), mean (SD)	5.47 (6.80)	4.87 (5.76)	8.73 (10.17)	0.0000
In-hospital LOS (Hrs), mean (SD) (including boarding time)	278.75 (420.32)	299.82 (441.11)	165.69 (255.82)	0.0000
In-hospital mortality, n (%)	520 (8.17)	424 (7.90)	96 (9.60)	0.072
In-hospital mortality within 48 h of admission, n (%)	94 (1.48)	62 (1.16)	32 (3.20)	0.000
Mortality within 28 days of discharge, n (%)	914 (14.36)	761 (14.18)	153 (15.30)	0.353
Mortality within 28 days of discharge (excluding in hospital death), n (%)	394 (6.19)	337 (6.28)	57 (5.70)	0.485
Readmitted within 7 days, n (%)	145 (2.28)	120 (2.24)	25 (2.50)	0.607
Readmitted within 28 days, n (%)	328 (5.15)	281 (5.24)	47 (4.70)	0.482
Discharge summary sent within two days of discharge, n (%)	4873 (76.54)	4217 (78.57)	656 (65.60)	0.000
Discharge summary sent within 7 days of discharge, n (%)	5538 (86.98)	4741 (88.34)	797 (79.70)	0.000
Discharged from Outlier Ward, n (%)	995 (15.63)	153 (2.85)	842 (84.20)	0.000
Admission decision made inside of working hours (0800 – 1800), n (%)	2771 (43.52)	2387 (44.48)	384 (38.40)	0.000

## Results

### Patient and admission characteristics

A total of 7070 patients admitted to FMC had hospital separation records that contained diagnoses of dementia and/or delirium according to the ICD-10-AM codes (Table 1) during the study period. These patients had a mean age of 81 years. The age for about 92.0% of these patients were more than 65 years (age range: 65–103). They represented 1.9% of the total patients admitted to the hospital, and 5.5% of those FMC patients aged 65 years and over who were admitted during the study period. About 90.0% of these patients (*n* = 6367) were either classified as ‘outlier’ patients (*n* = 1000; 15.7%) or as ‘inlier’ patients (*n* = 5367; 84.3%). The episode of care for about 98.0% (*n* = 6225) of the cohort was classified as acute.

Most of the ‘outlier’ and the ‘inlier’ patients were triaged in the ED and were assigned to one of the five ATS categories. About 8.0% (*n* = 506) of the cohort; mainly the ‘inlier’ patients were not triaged (ATS 6) and/or their priority was not assigned. This was likely due to being admitted as elective patients, having been transferred from another hospital for admission, or patients who followed a direct pathway to a specific ward such as an obstetrics/gynaecology admission.

The source of referral recorded were admission through the hospital’s ED as a casualty/emergency admission (50.2%; *n* = 3195), other or unknown referral (37.7%; *n* = 2403), referral from other private medical practice (2.6%; *n* = 166), referral from residential aged care facilities (4.5%; *n* = 288), inter-hospital transfer (1.8%; *n* = 114) and referral from the outpatient department (3.2%; *n* = 201). At FMC, those patients presenting to the ED that require admission are referred for admission by the ED doctors to an appropriate inpatient team (unit). Upon consultation with the unit’s consultant, some patients may be admitted directly to a unit after assessment by a doctor at a private practice, outpatient department or at another public hospital. These patients are admitted directly with no further input from the ED doctors unless the patient requires immediate treatment at the ED.

The top ten treated principal conditions recorded were: Urinary tract infection (5.5%), Pneumonia (5.4%), Pneumonitis due to inhalation of food and vomit (3.4%), Midcervical fracture of femur (2.9%), Unspecified dementia (2.8%), Delirium unspecified (2.7%), Delirium superimposed on Dementia (2.6%), Alzheimer’s disease (2.3%), Fracture of greater trochanter of femur (2.0%) and Syncope and collapse (1.9%). Although dementia and/or delirium were the principal diagnosis in 11.4% of the cohort but; Dementia, Delirium and Alzheimer’s disease were the primary condition treated only for about 10% of the cohort during their episode of care at the hospital. Treatment is performed after further examination of the patient; therefore there might be cases where the primary treatment offered may not be in alignment with the initial (principal) diagnosis recorded at the time of admission.

### Relationship between dependent variable and independent variables (risk factors and health system outcome measures)

#### ATS category

Based on univariate logistic regression analysis and after controlling for independent variables, the ‘outlier’ patients are more likely to be triaged under ATS 5 category when compared with the ‘inlier’ patients (OR: 2.519, 95%, CI: 1.271–4.994, *p* = 0.008). Patients triaged under ATS 5 category are considered to be less urgent and their clinical outcome are considered not to be significantly affected if treatment is delayed for up to two hours [22].

#### Charlson co-morbidity index

Mean Charlson co-morbidity index was 2.0; however, after adjusting for independent variables, the Charlson co-morbidity index for the ‘outlier’ patients is more likely to be higher than for the ‘inlier’ patients (OR: 1.059, 95% CI: 1.021–1.099, *p* = 0.002).

#### ED LOS

**Fig. 2:**
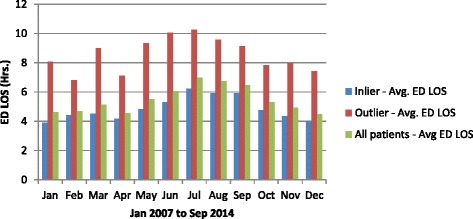
Monthly dementia and/or delirium average ED LOS

**Fig. 3:**
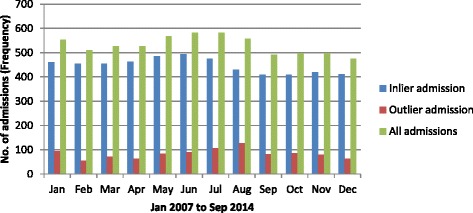
Monthly dementia and/or delirium admission

**Fig. 4:**
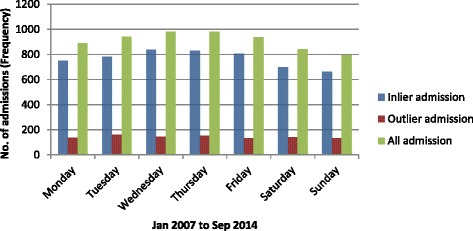
Daily dementia and/or delirium admission

**Fig. 5:**
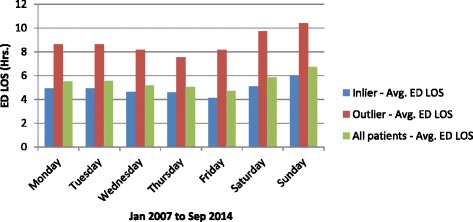
Daily dementia and/or delirium average ED LOS

During the study period, the average ED LOS was 5.5 h. ‘Outlier’ patients’ stay in the ED was 3.9 h longer than that of ‘inlier’ patients. For the ‘outlier’ patients, average ED LOS was persistently higher than the ‘inlier’ patients and persistently higher than the entire cohort (presented in Fig. 2). As expected the ED LOS worsened during the winter months for both ‘outlier’ and ‘inlier’ patients corresponding with the higher numbers of admissions (presented in Fig. 3). During the winter months of June, July and August the average ED LOS for ‘outlier’ patients versus ‘inlier’ patients were (10.0 cf. 5.3 h), (10.3 cf. 6.2 h) and (9.6 cf. 6.0 h) respectively (presented in Fig. 2). Regardless of seasonal variation, ED LOS for the ‘outlier’ patients remained higher than the ‘inlier’ patients. Additionally, despite the drop in admissions to the hospital on the weekend (presented in Fig. 4), the average ED LOS was higher than week days (presented in Fig. 5). The average ED LOS was 5.9 h on Saturday and 6.7 h on Sunday. The average ED LOS for ‘outlier’ patient versus ‘inlier’ patients were (9.7 cf. 5.9 h) on Saturdays and (10.4 cf. 6.0 h) on Sundays. After adjusting for independent variables, ‘outlier’ patients’ LOS in the ED continued to be prolonged; ‘outlier’ patients ED LOS doubled (OR: 1.068, 95% CI: 1.057–1.079, *p* = 0.000) when compared with ’inlier’ patients.

#### Inpatient LOS

**Fig. 6:**
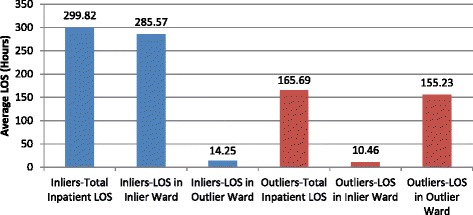
Average LOS comparison for Inliers and Outliers

The average inpatient LOS for those patients aged 65 years and over and coded with a dementia and/or delirium diagnosis was 260.4 h or 10.9 days. ‘Outlier’ patients’ inpatient LOS was 134.1 h or 5.6 days less than that of ‘inlier’ patients’. We confirmed that the overall longer inpatient LOS for the ‘inlier’ patients was not the result of our > =70% classification threshold. In other words, for the ‘inlier’ patients; LOS in outlier wards/s was only a small percentage of the overall inpatient LOS (presented in Fig. 6) and the prolonged inpatient LOS was not a consequence of extended stay in outlier wards/s whilst a bed in home-ward was being sourced. ‘Inlier’ patients only spent on average about 4.8% of their inpatient LOS in outlier ward/s and the ‘outlier’ patients only spent on average about 6.3% of their inpatient LOS in home-ward/s. After adjusting for independent variables, ‘outlier’ patients’ inpatient LOS continued to be shorter than ‘inlier’ patients. Therefore, ‘outlier’ patients are more likely to stay at the hospital for a shorter duration when compared with ‘inlier’ patients (OR: 0.998, 95% CI: 0.998–0.999, *p* = 0.000).

#### In-hospital mortality

About 8.2% of the entire cohort died during their episode of care at the hospital. The proportion of ‘outlier’ patients who died in the hospital within 48 h of admission and within 28 days of discharge was higher than the proportion of ‘inlier’ patients. However, only death within 48 h of admission was statistically significant between the ‘outlier’ and the ‘inlier’ patients. After controlling for all other independent variables, ‘outlier’ patients are more likely to be at risk of dying in the hospital within 48 h of admission (OR: 1.973, 95% CI: 1.158–3.359, *p* = 0.012) compared with ‘inlier’ patients showing inferior health systems outcome for the ‘outlier’ patients.

#### Discharge summary

A further finding demonstrates that discharge summaries were not sent within 2 days as required by the hospital for about 34% of the ‘outlier’ patients as opposed to about 21% of ‘inlier’ patients. After adjusting for the independent variables, ‘outlier’ patients are more likely to be at risk of not receiving their discharge summaries within 2 days of discharge from hospital (OR: 1.75, 95% CI:1.492–2.061, *p* = 0.000) when compared with inlier patients posing concerns for continuity of care.

### Nature of separation (destination after eventual hospital discharge)

The majority of the patients were discharged back to their usual place of residence prior to their hospital admission. A higher proportion of ‘outlier’ patients (17.6%) than ‘inlier’ patients (8.3%) were transferred to another hospital (up transfer) to continue their care (*p* = 0.000). Patients are up transferred from FMC to another hospital if they require specialised care not offered at FMC; such care may entail rehabilitation or palliative care. After adjusting for independent variables, ‘outlier’ patients are more likely to be up transferred (OR: 1.931, 95% CI = 1.559–2.391, *p* = 0.000) when compared with ‘inlier’ patients.

#### Sensitivity analysis

The results of a sensitivity analysis based on 100% threshold for the dependent variable reduced the eligible cohort to 18% instead of the 90% achieved with the > =70% threshold. About 59% (*n* = 750) observations were dropped because of collinearity and the model could not be fit with a number of the independent variables. The independent variables affected were ‘treatment priority’, ‘time spent boarding in the ED’, ‘in-hospital mortality within 48 h of admission’ and ‘mortality within 28 days of discharge’. As such the results of this paper are based on the > =70% threshold which was based on a model that was able to fit the important variables of interest. The characteristic of excluded patients as a result of applying the > =70% threshold is presented in Table 4. The results are within the expected range with about 64% (*n* = 452) of these patients spending more than half of their hospital stay in home-ward/s.

**Table 4:** Excluded patient characteristics

	Total (*n* = 703)
Demographics	
Age, mean (SD)	81.84 (11.01)
Charlson Index, mean (SD)	2 (2.11)
Female, n (%)	391 (55.62)
Principal Diagnosis coded with Dementia, n (%)	105 (14.94)
Treatment Priority	
ATS 1, n (%)	30 (4.27)
ATS 2, n (%)	156 (22.19)
ATS 3, n (%)	362 (51.49)
ATS 4, n (%)	131 (18.63)
ATS 5, n (%)	6 (0.85)
ATS 6, n (%)- *(Not triaged)*	0
Not assigned, n (%)	18 (2.56)
Episode of Care	
Acute, n (%)	691 (98.29)
Non-Acute, n (%)	12 (1.71)
Nature of Separation	
Home, n (%)	397 (56.47)
Other hospital – up transfer, n (%)	67 (9.53)
Nursing home or hostel, n (%)	56 (7.97)
In-hospital mortality, n (%)	81 (11.52)
Other hospital – down transfer, n (%)	94 (13.37)
Other types of discharge, n (%)	8 (1.14)
Time spent boarding in ED (Hrs), mean (SD)	8.25 (8.70)
In-hospital LOS (Hrs), mean (SD) (including boarding time)	234.01 (286.73)

## Discussion

The analyses of these data in this study demonstrates that the location of care of a patient with dementia and/or delirium diagnoses during an episode of inpatient care at a tertiary hospital can affect their health system outcomes. In this study, we found that the health system outcomes were inferior for the ‘outlier’ patients even after adjusting for confounding factors. The higher likelihood of ED presentations attributed to the aging population coupled with higher hospital admission rates for people with dementia [23] and that room transfers promote the development of delirium [16], calls for extra attention to be given when hospitals are deriving policies surrounding bed allocation to units predominantly responsible for the care of these patients. About 72% of our entire patient cohort was assigned to ATS 1 to ATS 3 categories; required urgent assessment by a clinician within the 30 min prescribed assessment time for ATS 3 patients [22]. However, the adjusted ATS category shows that the ‘outlier’ patients are more likely to be assigned to ATS 5 category compared to the ‘inlier’ patients. Furthermore, because of inability to provide information during admission, the likelihood of under triage is higher for cognitively impaired patients [24]. These ‘outlier’ patients could potentially spend up to 2 h or longer in the ED from the time of their triage to when the admission decision is made. Being acutely unwell and having to wait for up to 2 h or more for assessment may contribute to rapid functional decline of these ‘outlier’ patients. In the busy ED environment, especially when patients fall in the non-urgent treatment category, busy ED staff may not recognise the cognitive impairment in these patients. Not recognising cognitive impairment may lead to significant functional decline in elderly patients [25]. The ‘outlier’ patients are more likely to have a higher Charlson co-morbidity index indicating that these patients are a sicker cohort, as such not recognising the cognitive impairment may lead to further deterioration of their overall health status and cognitive ability. Co-morbidity burden has been associated with rapid cognitive decline especially in patients aged 65 and older diagnosed with Alzheimer’s disease [26].

The ‘outlier’ patients being sicker and their treatment priority in the ED categorised as less urgent will naturally contribute to prolonged ED LOS. Our study showed that ‘outlier’ patients’ ED LOS was longer than ‘inlier’ patients’. As expected the ED LOS was higher during busy periods (*e.g. winter months and weekend*) for the entire cohort and this increase in ED LOS was exacerbated for the ‘outlier’ patients. Moreover, the daily and monthly pattern of ED LOS for the ‘outlier’ patients was consistently higher than that of ‘inlier’ patients. The prolongation of ED LOS for the ‘outlier’ patients remained significant even after adjusting for independent variables. Less urgent ATS categories may partly contribute to the longer ED LOS for the ‘outlier’ patients. The less urgent triage category and its association with prolonged assessment time leading to eventual admission of a patient has been studied before on a group of GM patients at the same hospital [27]. Another study also reported that elderly patients with dementia were predictors of prolonged ED LOS [28].

The presence of ‘ED congestion’ during periods of high occupancy rates, where ED is incapacitated by patients waiting for unavailable beds, makes the likelihood of finding a home-ward even harder [27]. Less than half or 44.0% of the entire cohort managed direct admission to their home-ward during the study period raising concerns of severe bed shortage in home-wards for this dementia and/or delirium patients. Our study showed that about 16.0% of the entire cohort and the majority of the ‘outlier’ patients (84.0%) were discharged from an outlier ward without ever making it to a home-ward.

Despite the prolonged ED LOS, the adjusted inpatient LOS for the ‘outlier’ patients were shorter compared to the ‘inlier’ patients. This result did not come as a surprise as it was similar to the findings from an earlier study of GM patients at the same hospital [10], where the inpatient LOS was shorter in outlying patients. It may be debated that the presence of the Acute Medical Unit (AMU) facility at the hospital, which generally admits short-stay patients (LOS of 24 to 48 h), may have contributed to the shorter inpatient LOS for the ‘outlier’ patients, especially if the predicted short-stay patients are predominantly housed in AMU. We confirmed that AMU does not influence the findings of our study. Only about 27.0% of the ‘outlier’ patients were ever admitted in the AMU as opposed to 43.0% of the ‘inlier’ patients. It may also be argued that functional assessment and/or sub-acute rehabilitation may dictate that the ‘outlier’ patients move to a home-ward later in time and become classified as ‘inlier’ patients, thus increasing the overall LOS for the ‘inlier’ patients in this study. Our method of using the > =70% cut-off point to classify the ‘outlier’ and the ‘inlier’ patients in this study is robust and prevents this occurrence. At the end of their stay, the ‘inlier’ patients stayed 286 h (12 days) in home-ward and only 14 h in outlier ward/s on average. Whereas the ‘outlier’ patients stayed 155 h (6.50 days) in outlier ward/s and 10 h in home-ward on average. These data demonstrate that the prolonged inpatient LOS for ‘inlier’ patients was not as a consequence of a prolonged stay in outlier ward/s whilst a bed in home-ward is being sourced (presented in Fig. 6). Similar to the previous findings of other researchers [29] we do not equate a shorter LOS with improved quality of care for the ‘outlier’ patients; there is no difference in the readmission rate between ‘outliers’ and ‘inliers’. In-hospital mortality rate, the risk of readmission and timely dissemination of discharge summaries reflects quality of care.

Patient age and co-morbidity separately impose significant effects on in-hospital mortality, LOS and risk of readmission [15]. Interestingly, the risk of readmission was not significantly different between the ‘outlier’ and the ‘inlier’ patients. The adjusted risk of death within 48 h of admission was higher for the ‘outlier’ patients. For the entire cohort about 70.0% of deaths within 48 h of admission occurred in outlier wards. The higher mortality in the ‘outlier’ patient group may not only reflect the system of care provided but also that these patients maybe more acutely unwell. The higher mortality figures are a significant cause for concern if our data reflect admitting patients initially to outlier wards as a common phenomenon. What our study cannot tell us is if relocating these patients promptly to home-ward has a protective effect for mortality.

Furthermore, once discharged from the hospital, the discharge summaries containing information about an episode of hospital care are less likely to be sent to the primary health care practitioners within 2 days of discharge compromising the continuity of care for the ‘outlier’ patients. Timely dissemination of discharge summaries is essential for continuity of care and for better health system outcomes [30]. In addition to being clinically complex, the ‘outlier’ patients are more likely to be transferred to another hospital to continue with care that is not available at FMC making timely completion of discharge summaries for these patients even more critical.

### Strength and limitations

Although dementia coding in administrative datasets is known to be low, studies using administrative dataset and ICD codes have been validated as an important starting point on dementia prognosis [31]. Research demonstrates significant under-coding of dementia and of patients with cognitive deficits [8, 32]. A diagnosis of dementia may not be recorded in hospital data if the condition does not affect the resource usage or the provision of care during the hospital stay [33]. In this study the dementia and/or delirium patients were identified if a dementia and/or delirium diagnosis were present in a patient’s hospital admission data therefore the patients with dementia and/or delirium that affected their care were more likely to have been identified as having the condition. The study compares the health system outcomes of hospitalisation for ‘outlier’ versus ‘inlier’ patients of all patients diagnosed with dementia and/or delirium (e.g. all patients had the same diagnosis), therefore not faced with issues apparent when comparing population with and without the diagnosis. In addition, our method of outlier and inlier classification is dependent on the proportion of time spent inside or outside of a home-ward rather than using the more simplistic way of directly classifying those who stayed outside of their home-ward as outliers. However, a sensitivity analysis undertaken based on 100% threshold for the dependent variable, although under a more stringent definition of ‘outlier’ and ‘inlier’ status, did not allow for full investigation of all the covariates (independent variables) of interest. Furthermore, although spending the entire hospital stay in a home-ward would be an ideal situation for a patient; this is unusual and difficult to achieve in hospitals currently, especially when the hospital is operating at close to full capacity. The data clearly shows that the majority of patients stayed in both inlier and outlier wards.

Our study has several limitations. It is retrospective and observational, and has relied on information available in hospital administrative datasets and gave insight into health system outcomes. Further research is needed to detect differences in actual care practices and functional outcomes of the patients. About 38% of patients did not have a source of referral and being an ‘outlier’ was not associated with arriving in the ED without a referral as majority were ‘inlier’ patients (83.0%). This is one of the challenges of using administrative datasets for research purposes. The process of collecting information changes over time and largely dependent on reporting needs. Also, the data are derived from one hospital, albeit covering various units within that hospital. Although within FMC there was a variation in the coding practice of dementia and/or delirium patients over time, this work is an important foundation for further analysis of hospital outcomes for the not insignificant number of patients who have recognised cognitive impairment.

The work clearly needs replication elsewhere. Ethical and practical issues preclude a randomised intervention trial of the study of outlier patient status, but a prospective observational study would allow collection of robust clinical data currently not accessible from administrative datasets. Future studies should include data on intensity of allied health intervention offered to patients in home-wards versus outlier wards which benefit the patients but may invariably increase the LOS. Such a study would be of further interest if it incorporated comparison between two institutions, one of a ‘business as usual’ type and another with a holistic dementia care approach. Issues that could be studied in a prospective study may include, appropriateness and impact on LOS versus investigation performed during hospital stays, or the impact of decision making in relation to issues such as the need for behavioural management, delirium identification in ED and other dementia-specific care. Future research should also explore the effects of higher in-hospital mortality within 48 h of admission versus shorter LOS for the ‘outlier’ patients and the higher likelihood of hospital–up transfer versus shorter LOS for the ‘outlier’ patients which our current research is not able to answer.

## Conclusion

Our study compares the risk factors and health system outcomes (or consequences) for patients with dementia and/or delirium when these patients are housed in wards distant from their home-ward using a large cohort of patients. We have studied one aspect of the impact of the organisation of care provided to dementia and/or delirium patients in a busy tertiary hospital in Australia. The ward location of a patient’s care appears to have substantial impact on the health system outcomes; with prolonged ED LOS and the overall inpatient LOS reduced for the ‘outlier’ patients. Further research is needed to identify those factors that are influencing reduced inpatient LOS to ensure that any organisational benefits that might derive from a reduced LOS are not being obtained at the expense of less than optimal inpatient care. Outlying patients had higher Charlson co-morbidity index and had higher chance of dying in-hospital within 48 h of admission. Timely dissemination of discharge summaries was compromised for outlying patients. Discharged outlying patients were more likely to be transferred to another hospital to continue care at another hospital. Although, in some cases staying in outlier ward (e.g. no location shift) might be perceived to be better for the patient as it provides better continuity of care, our study shows that the health system outcome for the outlying patients was poor.

Better acute care for frail dementia and/or delirium patients may reduce the burden of hospital care overall and in turn reduce the rising cost of care attributed to dementia. Administrative data sets are widely used as a starting point in dementia research; therefore it is imperative to develop processes to improve dementia identification and coding in hospital administrative data.

## Abbreviations

ATS: Australian triage scale
CI: Confidence interval
ED: Emergency Department
FMC: Flinders Medical Centre
ICD-10-AM: International Classification of Diseases, Tenth Revision, Australian Modification
LOS: Length of Stay
OR: Odds ratio

## References

[1] Australian Institute of Health and Welfare 2012. Dementia in Australia. In: Cat. no. AGE 70. Canberra: AIHW; 2012.

[2] Australian Institute of Health and Welfare 2013. Australia’s welfare 2013. Canberra: AIHW; 2013.

[3] Korevaar JC, van Munster BC, de Rooij SE (2005). Risk factors for delirium in acutely admitted elderly patients: a prospective cohort study. BMC Geriatr.

[4] LoGiudice D, Watson R (2014). Dementia in older people: an update. Intern Med J.

[5] Sepulveda E, Franco JG, Trzepacz PT, Gaviria AM, Vinuelas E, Palma J (2015). Performance of the Delirium Rating Scale-Revised-98 Against Different Delirium Diagnostic Criteria in a Population With a High Prevalence of Dementia. Psychosomatics.

[6] Meagher DJ, Leonard M, Donnelly S, Conroy M, Saunders J, Trzepacz PT (2010). A comparison of neuropsychiatric and cognitive profiles in delirium, dementia, comorbid delirium-dementia and cognitively intact controls. J Neurol Neurosurg Psychiatry.

[7] Fick DM, Agostini JV, Inouye SK (2002). Delirium superimposed on dementia: a systematic review. J Am Geriatr Soc.

[8] Draper B, Karmel R, Gibson D, Peut A, Anderson P (2011). The Hospital Dementia Services Project: age differences in hospital stays for older people with and without dementia. Int Psychogeriatr.

[9] Australian Institute of Health and Welfare 2013. Dementia Care in Hospitals: costs and strategies 2013. Canberra: AIHW; 2013.

[10] Perimal-Lewis L, Li JY, Hakendorf PH, Ben-Tovim DI, Qin S, Thompson CH (2013). Relationship between in-hospital location and outcomes of care in patients of a large general medical service. Intern Med J.

[11] Santamaria JD, Tobin AE, Anstey MH, Smith RJ, Reid DA (2014). Do outlier inpatients experience more emergency calls in hospital? An observational cohort study. Med J Aust.

[12] Warne S, Endacott R, Ryan H, Chamberlain W, Hendry J, Boulanger C (2010). Non-therapeutic omission of medications in acutely ill patients. Nurs Crit Care.

[13] Sampson EL, Gould V, Lee D, Blanchard MR (2006). Differences in care received by patients with and without dementia who died during acute hospital admission: a retrospective case note study. Age Ageing.

[14] Buist MD, Jaffray L, Bell E, Hanna L, Weinstein P, Kumar S (2014). Utilisation of beds on the general medical unit by ‘non-acute medical’ patients: a retrospective study of incidence and cost in two Tasmanian regional medical hospital units. Intern Med J.

[15] Australian Institute of Health and Welfare 2012. Dementia in Australia 2012. Canberra: AIHW; 2012.

[16] Goldberg A, Straus SE, Hamid JS, Wong CL (2015). Room transfers and the risk of delirium incidence amongst hospitalized elderly medical patients: a case–control study. BMC Geriatr.

[17] Dusetzina SB, Tyree S, Meyer AM (2014). Linking Data for Health Services Research: A Framework and Instructional Guide [Internet].

[18] McDonald KM, Sundaram V, Bravata DM (2007). Closing the Quality Gap: A Critical Analysis of Quality Improvement Strategies (Vol. 7: Care Coordination).

[19] Charlson ME, Pompei P, Ales KL, MacKenzie CR (1987). A new method of classifying prognostic comorbidity in longitudinal studies: development and validation. J Chronic Dis.

[20] Quan H, Sundararajan V, Halfon P, Fong A, Burnand B, Luthi JC (2005). Coding algorithms for defining comorbidities in ICD-9-CM and ICD-10 administrative data. Med Care.

[21] Thabane L, Mbuagbaw L, Zhang S, Samaan Z, Marcucci M, Ye C (2013). A tutorial on sensitivity analyses in clinical trials: the what, why, when and how. BMC Med Res Methodol.

[22] Australasian College for Emergency Medicine. Guidelines on the Implementation of the Australasian Triage Scale. URL:https://www.acem.org.au/getattachment/4320524e-ad60-4e7c-a96d-bdf90cd7966c/G24-Implementationofthe-Australasian-Triage-Scal.aspx. Accessed 01 Oct 2015.

[23] Phelan EA, Borson S, Grothaus L, Balch S, Larson EB (2012). Association of incident dementia with hospitalizations. Jama.

[24] Lamantia MA, Stewart PW, Platts-Mills TF, Biese KJ, Forbach C, Zamora E (2013). Predictive value of initial triage vital signs for critically ill older adults. West J Emerg Med.

[25] Hsiao FY, Peng LN, Wen YW, Liang CK, Wang PN, Chen LK (2015). Care needs and clinical outcomes of older people with dementia: a population-based propensity score-matched cohort study. PLoS One.

[26] Aubert L, Pichierri S, Hommet C, Camus V, Berrut G, de Decker L (2015). Association between comorbidity burden and rapid cognitive decline in individuals with mild to moderate Alzheimer’s disease. J Am Geriatr Soc.

[27] Perimal-Lewis L, Ben-Tovim DI, Li JY, Hakendorf PH, Thompson CH (2014). Emergency department lengths of stay: characteristics favouring a delay to the admission decision as distinct from a delay while awaiting an inpatient bed. Intern Med J.

[28] Brick C, Lowes J, Lovstrom L, Kokotilo A, Villa-Roel C, Lee P (2014). The impact of consultation on length of stay in tertiary care emergency departments. Emerg Med J.

[29] Sampson EL, Blanchard MR, Jones L, Tookman A, King M (2009). Dementia in the acute hospital: prospective cohort study of prevalence and mortality. Br J Psychiatry J Ment Sci.

[30] Li JY, Yong TY, Hakendorf P, Ben-Tovim D, Thompson CH (2013). Timeliness in discharge summary dissemination is associated with patients’ clinical outcomes. J Eval Clin Pract.

[31] van de Vorst IE, Vaartjes I, Sinnecker LF, Beks LJ, Bots ML, Koek HL (2015). The validity of national hospital discharge register data on dementia: a comparative analysis using clinical data from a university medical centre. Neth J Med.

[32] Cummings E, Maher R, Showell CM, Croft T, Tolman J, Vickers J (2011). Hospital coding of dementia: is it accurate?. HIM J.

[33] Australian Institute of Health and Welfare (2012). Deriving key patient variables: A technical paper for the Hospital Dementia Services project.

